# 肺粘膜相关淋巴组织边缘区B细胞淋巴瘤的临床病理分析

**DOI:** 10.3779/j.issn.1009-3419.2011.05.12

**Published:** 2011-05-20

**Authors:** 勃江 陈, 俊 高, 源 唐, 尚福 张, 为民 李, 静 曾

**Affiliations:** 1 610041 成都，四川大学华西医院呼吸内科 Department of Respiratory Medicine, West China Hospital of Sichuan University, Chengdu 610041, China; 2 610041 成都，四川大学华西医院病理科 Department of Pathology, West China Hospital of Sichuan University, Chengdu 610041, China

**Keywords:** 肺肿瘤, 淋巴瘤, 诊断, 预后, Lung neoplasms, Lymphoma, Diagnosis, Prognosis

## Abstract

**背景与目的:**

肺粘膜相关淋巴组织边缘区B细胞淋巴瘤（pulmonary marginal zone B-cell lymphoma of mucosa-associated lymphoid tissue, PMZL-MALT）极少见，易误诊。本文旨在总结该病特征，以提高临床医生的认识水平。

**方法:**

回顾性分析四川大学华西医院2008年11月-2010年11月确诊的7例PMZL-MALT患者的临床病理资料。

**结果:**

女性5例，男性2例；中位年龄62岁（37岁-74岁）。以咳嗽、咳痰为主要症状（6例）；CT多表现为肺部实变影（6例），误诊为肺炎。纤维支气管镜是主要的侵入性检查方法（6例）；7例患者肿瘤细胞免疫表型均为CD20和CD79a细胞膜弥漫强阳性表达，CD3ε、CD5、CyclinD1、CD10、Bcl-2和CD30不表达，Ki-67指数 < 10%；6例*IgH*基因重排分析可见克隆性条带。3例患者予以COP方案（环磷酰胺、长春新碱、泼尼松）化疗，其中1例加利妥昔单抗（RCOP）；1例予以CHOP方案（环磷酰胺、阿霉素、长春新碱、泼尼松）化疗；1例因糖尿病血糖控制不佳予以CTX（环磷酰胺）化疗；2例拒绝化疗。随访至2010年12月2日，仅1例既往有慢性阻塞性肺疾病史且未化疗者在确诊12个月后因呼吸衰竭死亡，其余患者的病情控制可。

**结论:**

PMZL-MALT患者的临床表现无特异性，组织病理学检查是确诊的唯一手段，患者的预后较好。

## Abstract (en)

**Background and objective:**

As a rare disease, pulmonary marginal zone B-cell lymphoma of mucosaassociated lymphoid tissue (PMZL-MALT), is often misdiagnosed. The aim of this study is to summarize the clinical and pathological features of this disease and improve the awareness of doctors.

**Methods:**

Seven cases (female 5, male 2) diagnosed of PMZL-MALT in West China Hospital between November 2008 and November 2010, were analyzed retrospectively, including their symptoms, radiological findings, pathological examinations, treatment and prognosis.

**Results:**

The median age of the patients were 62 years old (range 34-79 years). Six patients suffered from cough and sputum. Pulmonary consolidation was the most frequent manifestation, leading a misdiagnosis of pneumonia with CT examinations. Pathological diagnosis was obtained via fiberoptic bronchoscopy in six patients and percutaneous pulmonary biopsy for the rest one. In the seven cases, immunohistochemical results showed CD20(+), CD79a(+), while CD3 epsilon(-), CD5(-), CyclinD1(-), CD10(-), Bcl-2(-) and CD30(-). Additionally, the expression of Ki-67 was below 10%. Further PCR analysis showed evidence of immunoglobulin heavy chain gene rearrangement in tissues from six subjects. Based on the disease location and patients' wishes, compared with two cases just receiving symptomatic treatments, the other five ones took in chemotherapies.

**Conclusion:**

Since there were no specific clinical features for patients of PMZL-MALT, histopathological examination was the only effective means to confirm the diagnosis.

肺粘膜相关淋巴组织边缘区B细胞淋巴瘤（pulmonary marginal zone B-cell lymphoma of mucosa-associated lymphoid tissue, PMZL-MALT）发病率低，易误诊。现将四川大学华西医院近2年收治的7例PMZL-MALT分析报道如下，以期提高临床医生对该病的认识水平。

## 1 材料与方法

2008年11月-2010年11月我院活体组织检查确诊4, 769例非霍奇金淋巴瘤，其中肺非霍奇金淋巴瘤32例，而PMZL-MALT仅9例，7例PMZL-MALT患者有完整临床、病理资料。随访至2011年12月2日，详细分析其临床症状、胸部影像学改变、组织病理学检查、治疗及预后等，总结该病的临床病理特征。

7例标本均用4%中性甲醛固定，石蜡包埋，切片厚约4 μm，行HE染色；选用CD20、CD3ε、CD5、CyclinD1、CD10、Bcl-2、CD30、Ki-67指数标志物等行免疫组织化学染色（SP法）。试剂均购自福州迈新生物技术开发有限公司，操作按说明书进行。以已知阳性组织卷切片作阳性对照，PBS替代一抗作空白对照。PCR引物由我院病理科分子病理室合成，阳性对照为已确诊的B细胞性淋巴瘤患者的*IgH*重排阳性组织，阴性对照为反应性增生的淋巴组织。

## 2 结果

### 2.1 临床资料

7例PMZL-MALT患者女性多见；中位年龄62岁。首发症状以咳嗽、咳痰为主；1例无明显临床症状，于头皮溃疡型基底细胞癌手术前常规胸片检查发现。病程3个月-35个月，平均18个月，其中5例 > 12个月。所有患者均未扪及全身浅表淋巴结肿大。胸部CT检查病灶多位于右肺，尤其是右肺中叶；多表现为大片实变影（图 1），误诊为肺炎。纤维支气管镜是主要的侵入性检查方法，局部粘膜充血、肿胀，呈颗粒状，严重时可出现管腔狭窄（图 2），甚至闭塞。所有病例经组织病理学检查，均符合WHO淋巴造血组织肿瘤分类新标准^[1]^。除1例无明显症状外，其余6例患者在出现症状后3周-24个月确诊，平均6.5个月。确诊后2例患者拒绝化疗，仅对症处理；1例因糖尿病血糖控制不理想行CTX（环磷酰胺）化疗；4例予复合方案化疗。随访至2010年12月2日，除1例未化疗者[74岁，既往有慢性阻塞性肺疾病（chronic obstructive pulmonary disease, COPD）史]在确诊后12个月因呼吸衰竭死亡外，余患者一般情况较好，症状控制可。临床资料详见表 1。

**1:**
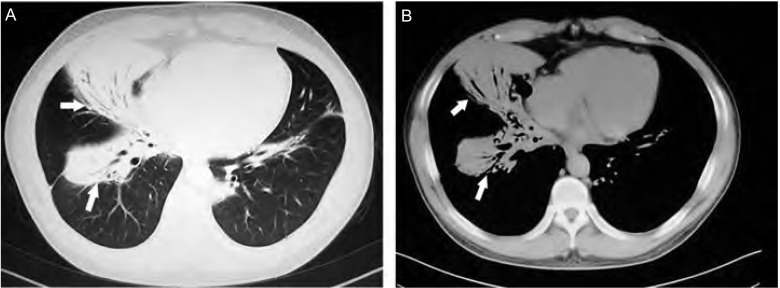
胸部CT显示右肺中叶大片实变影伴支气管气相征（箭头所指）。A：肺窗；B：纵隔窗 Extensive consolidation with air bronchogram in the middle lobe of right lung on CT scans (Arrow). A: pulmonary window; B: mediastinum window

**2:**
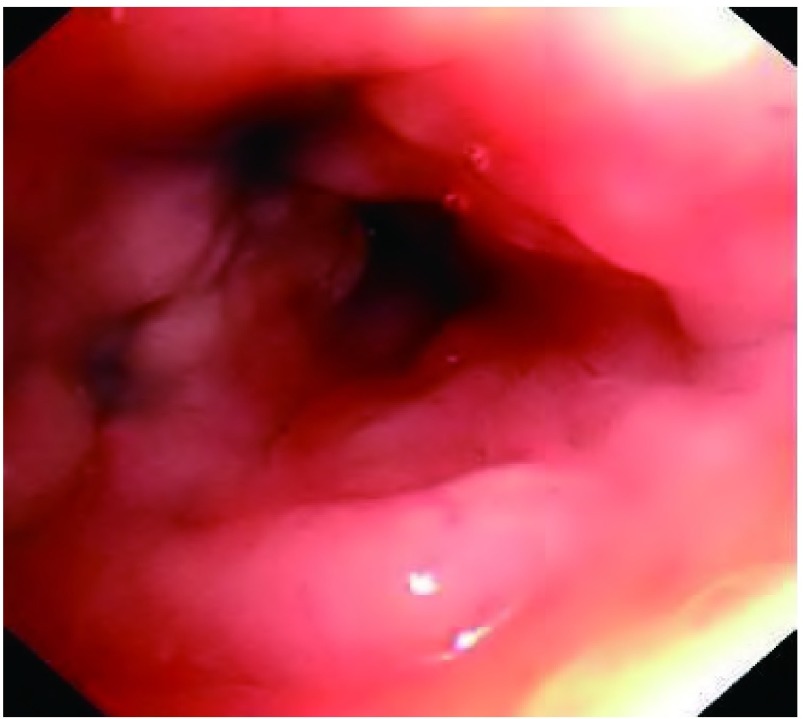
纤维支气管镜检查。右肺中叶支气管黏膜肿胀、充血，开口狭窄 Fiberoptic bronchoscopy. Swelling and congestion of the right middle lobar bronchial mucosa, with a narrowing opening

**1:** 7例PEMZL- MALT患者临床资料 Clinical characteristics of the seven patients of PEMZL- MALT

Case	Gender	Age (year)	Symptoms	Past medical history and personal history	Chest CT findings		Primary diagnosis	Invasive examination methods	Duration of disease /months	Delay for diagnosis /months	Treatment	Status
					Location	Characteristics						
1	Female	37	Cough Sputum Chest pain Fever	None	Right upper lobe; Right middle lobe	Consolidation with air bronchogram	Pneumonia	Fiberoptic bronchoscopy	26	9	COP	Survival
2	Male	39	Cough Sputum Chest pain Fever	None	Right middle lobe; Both lower lobes	Consolidation	Pneumonia	Fiberoptic bronchoscopy (Four times)	35	24	R-COP	Survival
3	Female	54	Cough Sputum Fever	None	Right middle lobe	Consolidation with air bronchogram	Pneumonia, Tuberculosis	Fiberoptic bronchoscopy	6	1	None	Survival
4	Female	62	Cough Sputum Dyspnea Chest pain	Diabetes mellitus	Left upper lobe	Mass (5.1 cm×5.8 cm)	Lung cancer	Percutaneous pulmonary biopsy	18	0.75	CTX	Survival
5	Male	65	Cough Sputum Dyspnea	Smoking (10 pack^*^ Years)	Right upper lobe	Consolidation with left pleural effusion	Pneumonia, Tuberculosis	Fiberoptic bronchoscopy	3	1.5	CHOP	Survival
6	Female	67	None	Basal cell carcinoma	Right upper lobe; Right middle lobe	Consolidation with air bronchogram	Pneumonia	Fiberoptic bronchoscopy	23	0	COP	Survival
7	Female	74	Cough Sputum Dyspnea Fever	COPD	Right middle lobe	Consolidation	Pneumonia	Fiberoptic bronchoscopy	15	3	None	Died
COP: cyclophosphamide, vincristine and prednisolone; R-COP: rituximab added to COP; CHOP: cyclophosphamide, doxorubicin, vincristine and prednisolone.												

### 2.2 病理特点

1例胸腔积液患者胸水中查见较多淋巴细胞。7例病变组织病理学检查均以小淋巴样细胞弥漫性浸润为特征。肿瘤细胞主要由“中心细胞样细胞”构成，体积较小，核轻度不规则，核仁不明显，染色质中等致密，细胞质较少，浅染或空亮；同时伴有淋巴细胞样细胞、淋巴浆细胞和单核细胞样B细胞等（图 3）。肿瘤细胞常广泛浸润支气管、细支气管粘膜和肺泡上皮，形成“淋巴上皮病损”。瘤细胞免疫表型均为CD20、CD79a细胞膜弥漫强阳性表达，CD3ε、CD5、CyclinD1、CD10、Bcl-2和CD30均不表达，Ki-67指数 < 10%（图 4）；采用PCR检测*IgH*基因表达，6例患者可见克隆性重排条带（图 5）。

**3:**
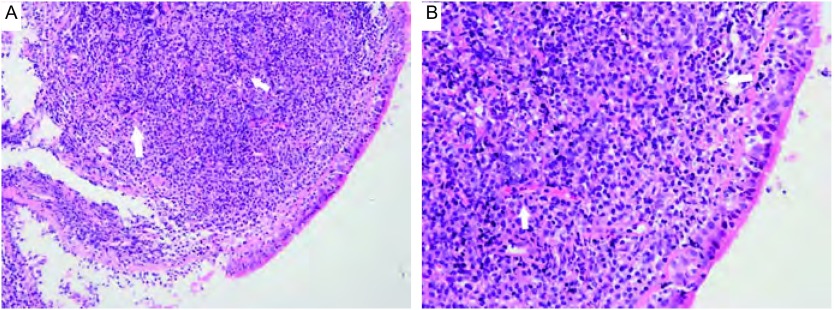
组织病理学检查。A：支气管黏膜中弥漫性小淋巴样细胞浸润，形成“淋巴上皮病损”（箭头所指）（HE，×100）；B：大量中心细胞样细胞。细胞体积较小，核轻度不规则，细胞质较少（箭头所指）（HE，×200） Histopathology. A: Small lymphocytes distributed diffusely in bronchial mucosa, and formatted the lymphoepithelial lesions (Arrow)(HE, ×100); B: Lots of centrocyto-like cells. Tumor cells were small, with slightly irregular nucleus and little cytoplasm(Arrow) (HE, ×200)

**4:**
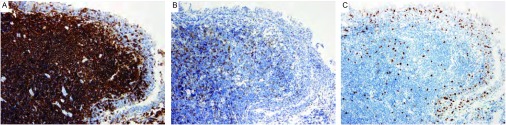
免疫组织化学染色（SP, ×200）。A：肿瘤细胞膜CD20弥漫强阳性表达（棕黄色颗粒）；B：肿瘤细胞不表达CD3*ε*。在CD20染色的同一部位行CD3*ε*染色，可见CD20弥漫强阳性表达的肿瘤细胞不表达CD3*ε*；图中棕黄色颗粒为正常T细胞；C：肿瘤细胞Ki-67指数 < 10%（棕黄色颗粒） Immunohistochemistry (SP, ×200). A: Positive immunoreaction for CD20 in the plasma membrane of tumor cells (brown particles); B: Negative immunoreaction for CD3*ε* in tumor cells; CD3 epsilon staining for the tissues with strong expression of CD20 showed negative results of CD3 epsilon; Brown particles indicated normal T cells; C: The expression of Ki-67 in tumor cells was below 10% (brown particles)

**5:**
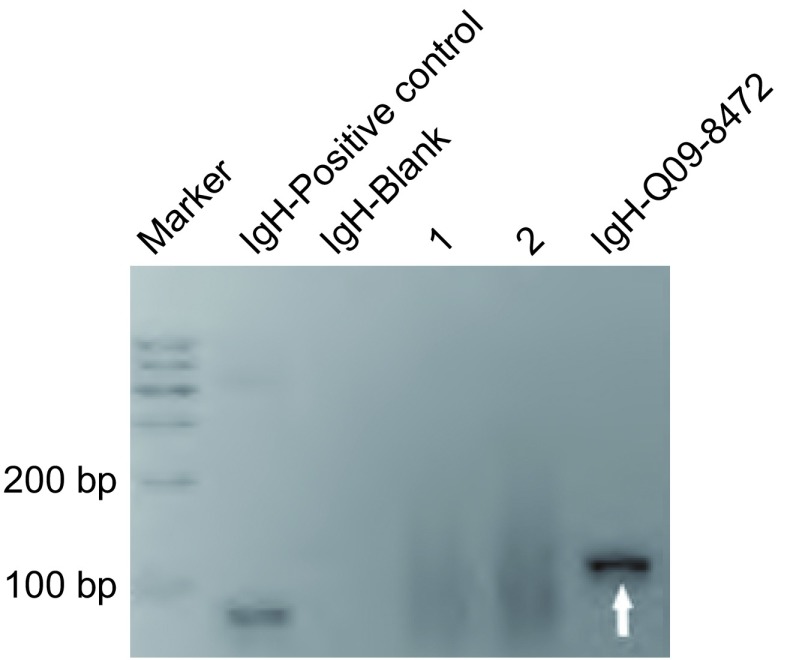
PCR法检测*IgH*基因重排分析110 bp处见克隆性重排条带（箭头所指）。图中“1、2”号条带为临床工作中同时检测的其它病例标本 Clonal rearrangement band of IgH detected by PCR analysis (Arrow). The bands marked with " 1" and " 2" were specimens of other cases, which were detected simultaneously with that from the targeted patient discussed in the text

## 3 讨论

PMZL-MALT的发病机制不清，一般认为由淋巴组织在抗原作用下克隆性增生所致，但病因尚不确定^[2]^。受发病率低、病例数少等因素限制，目前认为本病与性别的关系不明，不同报道差异较大，但一般均认为该病多见于中老年人^[3-5]^。该病恶性程度较低，患者的病程较长，临床表现取决于病变的部位和范围。当增生的淋巴细胞浸润支气管壁时，患者多出现咳嗽、咳痰、胸闷、气促等症状，可伴低热、消瘦等全身不适。由于管壁受累、气道不同程度狭窄、局部或全身防御力下降，部分患者可合并感染，出现畏寒、高热、咳脓痰等；但另有部分患者病程中可无任何不适，仅在体检时偶然发现^[4, 6]^。本组病例中上述各种表现均有，但以咳嗽、咳痰最为常见，应引起临床医生重视。

影像学异常是提示PMZL-MALT的重要线索，可分为实变型、肿块型、结节型和混合型^[5, 7]^。以实变型最常见，范围一般较大，可累及一个或多个肺叶，支气管气相是最有价值的特征，易误诊为肺炎。肿瘤细胞沿气道粘膜浸润性生长致气道不完全性阻塞是其主要病理学基础。肿块型常表现为单个直径≥2 cm的团块影，易与肺癌混淆；结节型则以多个 < 2 cm的大小不等结节影多见，散在分布。另外，少数病例表现为肺间质性改变，形成弥漫性网状结构；极少数还可出现胸腔积液^[3, 5]^，可能与淋巴管和血管周围粘膜相关淋巴组织增生，影响淋巴液、血液回流，增加淋巴管、血管壁通透性等因素有关。由于影像学改变无特异性，易致误诊，因此，当患者诊断不明时，应积极进行进一步检查以明确诊断。

本组7例患者的病程为3个月-35个月（也可能更长），平均18个月，其中5例 > 12个月，证实了本病的惰性特点。但患者从出现症状到确诊平均延迟6.5个月，多数患者首诊时诊断为肺炎或肺结核，并予以相应治疗。虽然如前所述，在PMZL-MALT的基础上，也可能合并感染，但基础疾病的存在势必影响抗感染治疗的效果。因此，当临床医生诊断肺部感染时，应当警惕是否存在其它病变，尤其是当患者对治疗反应不佳时，更应该考虑到有其它未知疾病，包括本病的可能，此时应该采取侵入性方式获得病变组织送病理活检诊断，以尽可能避免漏诊和误诊。

PMZL-MALT是以淋巴细胞克隆性增生为特点，因此组织病理学检查是诊断的金标准。活检组织主要通过手术、经皮肺穿刺、纤维支气管镜等侵入性方法获得；而痰脱落细胞检查多为阴性，这与肿瘤细胞的粘附性较好有关。但另有学者认为，该病病灶多位于段以下支气管，且肿瘤细胞在支气管粘膜下浸润性生长，因此纤维支气管镜在本病的检查中价值亦很有限^[3, 8]^。本研究除1例左上肺肿块由经皮肺穿刺活检外，其余患者均行纤维支气管镜活检，且5例在首次检查时获得阳性结果，另1例经过4次检查后确诊。事实上，该患者在第二次检查时已发现核异质细胞，但终因获得的病变组织少，取材不理想而未能确诊。因此，是否应该否认纤维支气管镜在本病中的检查价值尚待探讨，病变部位可能是选择检查方法的重要依据。

显微镜下，PMZL-MALT细胞可呈结节状或弥漫分布。低倍镜观察时，肿瘤细胞一般分布不均一，常见浆细胞或淋巴浆细胞^[9]^；高倍镜时，各种分化状态的肿瘤细胞则分散分布，较少出现条索、串珠或巢状结构^[9]^。作为一种惰性小B细胞性肿瘤，免疫组织化学染色是确诊与鉴别诊断的重要方法。本组7例病变组织均行免疫组织化学染色检测，其肿瘤细胞均呈CD20和CD79a弥漫强阳性表达，CD3ε、CD5、CyclinD1、CD10、Bcl-2和CD30均不表达，Ki-67指数 < 10%，有助于PMZL-MALT的病理学诊断。部分患者可有淋巴细胞免疫球蛋白重链的基因重排^[6, 9]^。本组病例中有6例患者均检出了IgH重排条带，而另1例阴性结果者则可能与引物选择、肿瘤细胞比例、标本质量等因素有关。另有报道约1/3的PMZL-MALT患者出现染色体异常，主要包括t（11, 18）、（q21, q21）和3号染色体三体型（+3）^[2, 9]^。

PMZL-MALT的恶性程度低，患者的5年及10年生存率分别可达90%和72%^[6, 10]^。因此，是否应对其进行积极的手术、化疗或放疗等处理，尚存争议。有学者推荐对局限性病灶进行手术切除，以防止少部分病变恶性演进；对弥漫性病变则应进行化疗，包括单药治疗和联合治疗两种方法。前者包括氟达拉滨、环磷酰胺、硫唑嘌呤、类固醇、利妥昔单抗等；后者如COP方案、CHOP方案、R-CHOP方案等^[4, 6]^。以上几种治疗方法的效果，目前尚未经循证医学研究论证。本组病例中，仅1例未化疗者因合并COPD，于诊断12个月后死于呼吸哀竭，其余病例均存活。

综上所述，PMZL-MALT患者的临床表现无特异性，临床医生应提高对本病的认识水平，适时地通过适当的侵入性检查进行组织病理学、免疫组织化学染色及基因重排分析是降低误诊率的唯一有效方法，患者的预后较好。
